# Tuning the Voices of a Choir: Detecting Ecological Gradients in Time-Series Populations

**DOI:** 10.1371/journal.pone.0158346

**Published:** 2016-07-28

**Authors:** Allan Buras, Marieke van der Maaten-Theunissen, Ernst van der Maaten, Svenja Ahlgrimm, Philipp Hermann, Sonia Simard, Ingo Heinrich, Gerd Helle, Martin Unterseher, Martin Schnittler, Pascal Eusemann, Martin Wilmking

**Affiliations:** 1 Institute of Botany and Landscape Ecology, Ernst-Moritz-Arndt-Universität, Soldmannstraße 15, 17487 Greifswald, Germany; 2 GFZ German Research Centre for Geosciences, Section 5.2, Telegrafenberg, 14473 Potsdam, Germany; University of Colorado, UNITED STATES

## Abstract

This paper introduces a new approach–the Principal Component Gradient Analysis (PCGA)–to detect ecological gradients in time-series populations, i.e. several time-series originating from different individuals of a population. Detection of ecological gradients is of particular importance when dealing with time-series from heterogeneous populations which express differing trends. PCGA makes use of polar coordinates of loadings from the first two axes obtained by principal component analysis (PCA) to define groups of similar trends. Based on the mean inter-series correlation (rbar) the gain of increasing a common underlying signal by PCGA groups is quantified using Monte Carlo Simulations. In terms of validation PCGA is compared to three other existing approaches. Focusing on dendrochronological examples, PCGA is shown to correctly determine population gradients and in particular cases to be advantageous over other considered methods. Furthermore, PCGA groups in each example allowed for enhancing the strength of a common underlying signal and comparably well as hierarchical cluster analysis. Our results indicate that PCGA potentially allows for a better understanding of mechanisms causing time-series population gradients as well as objectively enhancing the performance of climate transfer functions in dendroclimatology. While our examples highlight the relevance of PCGA to the field of dendrochronology, we believe that also other disciplines working with data of comparable structure may benefit from PCGA.

## Introduction

Various environmental disciplines deal with time-series populations derived from individuals. Individuals in the presented context may be living organisms or–in a more abstract sense–devices measuring the same parameter but at different locations. For reasons of simplicity, we here focus on the case of dendrochronological data. There, populations are forests (the ‘choir’) consisting of individual trees (the ‘voices’) which tree-ring parameters are considered proxies for tree performance and its driving mechanisms (the ‘song’).

Dendrochronological datasets typically consist of tree-ring parameter time series (e.g. ring-widths) which originate from at least 10 individuals investigated at one site [1, 2]. Standard data preparation comprises cross-dating of individuals [1] to assure common year to year growth variability which can be expressed by high Gleichlaeufigkeit (glk) [2, 3]. Mean inter-series correlation (rbar) is used to express shared long-term growth variance, which in the so-called expressed population signal (EPS) takes into account the number of samples contributing to rbar [4]. If these statistics reach fairly high values it is assumed that trees react to a common driver and can be used as proxies for this common signal [1, 4]. In such case single tree-ring parameter series are often averaged to a so-called master chronology as representation of the whole stand [1]. The idea behind this treatment is to enhance the strength of an assumed common signal as each single tree–due to its specific life history–has an individual noise distorting a common signal [1]. It has been shown that building master chronologies evens out individual noises which often is reflected by correlations between master chronologies and their dominant growth driver being higher than the majority of individual tree correlations [5].

Although being very efficient, this standard treatment largely neglects individualistic growth response of trees. Amongst others, [6] have shown that trees from one population may express common year-to-year variability (high glk) but different long-term (decadal to centennial) growth trends (low rbar). This behaviour has been reported from North America [7–10], Europe [11, 12], Central Asia [13, 14], and several sites around the circumpolar North [15]. If differing growth trends exist within one stand, master chronology building will rather decrease than increase the strength of correlations with growth drivers. In this context individual growth response has been discussed as possible cause of the so-called divergence effect [16] which is the apparent inability of tree-ring based climate reconstructions to capture recent warming trends [17, 18]. Although hypotheses exist (e.g. genetics, micro-topographical variations), the causes of differing growth trends have not been identified wherefore it is necessary both in dendroecological and dendroclimatological context to improve our understanding.

Many studies investigating growth divergence (as those mentioned above) defined so-called responder chronologies. Such chronologies consist of groups of trees with shared short- and long-term variance within each group but different long-term trends among groups. Thus, in contrast to typical master chronologies, which represent the average growth over all trees within one population, different responder chronologies represent groups of trees with differing long-term growth trends from the same population. To delimit groups of trees according to their specific growth trends, some studies used correlations of individual trees with climate [7, 8, 13, 14, 15, 19, 20], while others based their groups on average growth differences between two periods [21, 22] or absolute growth trends [9–12].

Although these studies showed convincing evidence that divergent growth trends within populations exist, the methods applied have their drawbacks. For instance, defining responder groups based on their climate correlations and then using the corresponding responder chronologies for climate reconstruction is considered ‘cherry picking’ [6]. A weakness specific to approaches using absolute growth differences or linear growth trends is that they do not cover specific scenarios. For instance, two tree-ring parameter series may express simoultaneously contrasting positive and negative growth anomalies, which cannot be detected by the aforementioned approaches. To overcome these problems, methods are needed which rank each tree-ring series according to their position along an inter-series correlation gradient inherent to the population and independent from the period selected. A respective tool theoretically should be capable of the following three applications, which will be referred to throughout the manuscript:

1. The identification of ecological gradients within the population, which for instance may be caused by population-internal (in case of tree-rings: age, competition, genotype, etc.) and/or population-external (soil properties, micro-climate, phytobiome, etc.) variability.
2. Explaining population gradients by statistically relating them to the aforementioned internal and external variables, this giving further insight into the mechanisms behind diverging growth trends. This application relies very much on the performance of I wherefore it will hold if I is fulfilled.
3. Defining responder chronologies on the basis of population gradients in the context of climate transfer function, to enhance signal strengths for the respective responder chronologies in comparison to the overall population master chronology.

First approaches to tackle these problems have been undertaken using Principal Component Analysis (PCA) [23] or Hierarchical Cluster Analysis (HCA) [20]. Although these approaches were able to clearly separate among responder groups (application III), they still lack the ability to express continuous population gradients as they result in categorical groups (Principal Component groups in [23] and clusters in [20]), thus not being capable of I and II.

In this context, we here introduce the Principal Component Gradient Analysis (PCGA). As in [23], PCGA is based on PCA but with the difference that it combines information from the first two PCA axes. Using polar coordinates of axes loadings PCGA defines a continuous population gradient. PCGA thus is a contribution to individualistic treatment of time-series within populations. As we will show, the resulting population gradients can be used to detect and explain the underlying mechanisms of ecological gradients, thereby meeting I and II. Furthermore, PCGA allows for defining responder groups to enhance the strength of common signals in terms of dendro-climatological reconstructions (III).

## Material and Methods

### 2.1. Data

#### 2.1.1. Pseudo-populations

As the aim of this paper is not only to introduce but also validate a new statistical approach we decided to generate artificial populations of ring-width series (RWS). These artificial populations we call pseudo-populations (PP). The advantage of PP is that they can be designed such that population gradients as well as dominant population signals of respective responder groups are known. A statistical method may be considered valid if it is able to correctly determine gradients within various PP and by this is able to correctly define responder groups which allow for an enhancement of signal correlations.

To explore a multitude of theoretically occurring scenarios, we altogether generated four PP. The general idea of PP generation was to take a single (for PP1) or two (PP2-PP4) RWS as underlying population signal(s) to generate 1000 RWS which all originate from these one or two RWS. To have a more realistic representation of tree-ring data, signals were taken from unpublished RWS-data of Scots Pine (*Pinus sylvestris* L.) growing on the Darß Peninsula, NE Germany. To remove any inherent trends we detrended the RWS using a ‘smoothing spline’.

**Fig 1:**
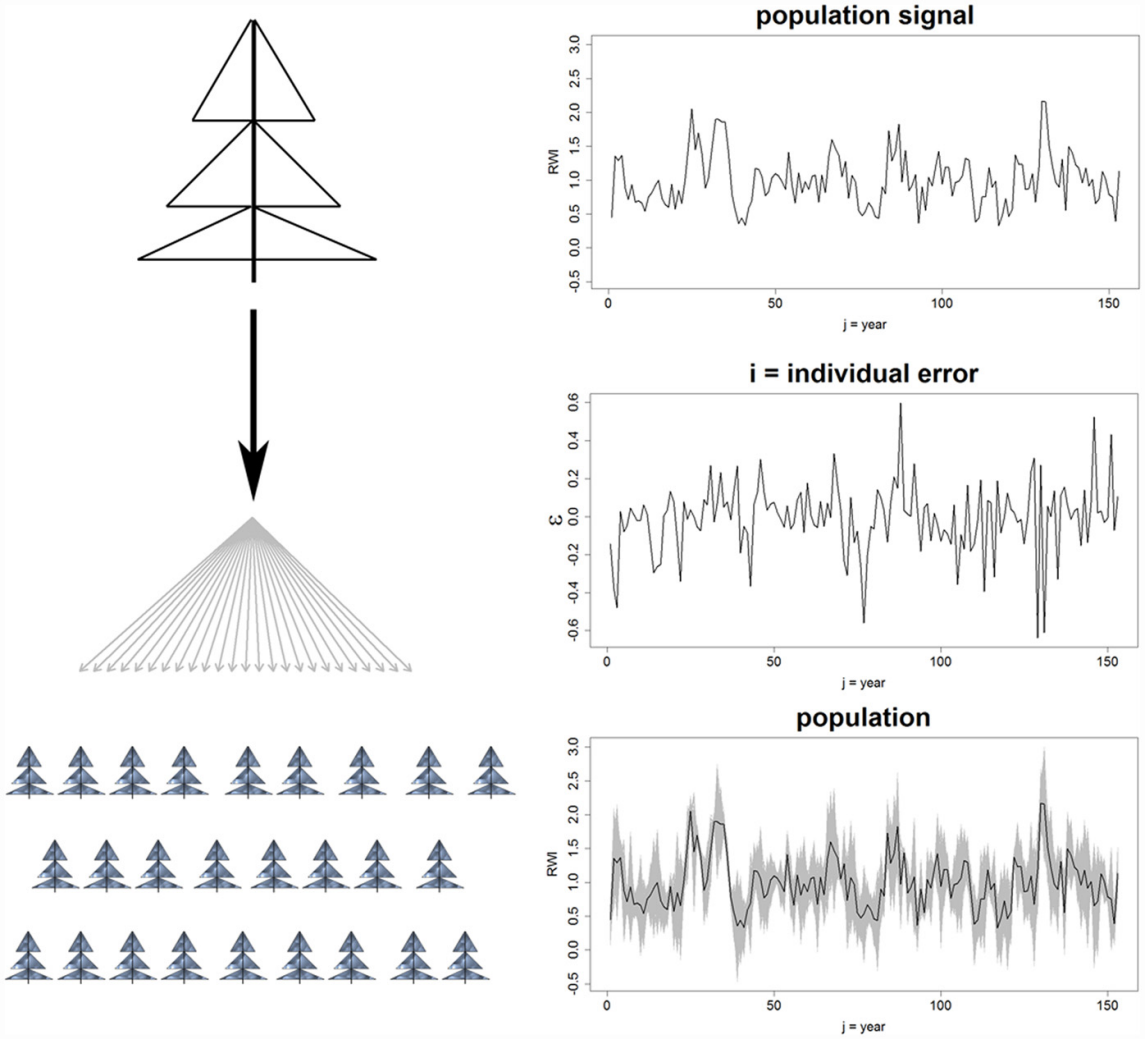
Generation of a homogeneous pseudo-population. From one Scots Pine ring-width series, 1000 pseudo-trees are generated by adding 1000 different white noises to this particular series.

For PP1 –a homogeneous PP which contained no clear population gradient but only individual-specific noises–we replicated one Scots Pine RWS 1000 times and added to each RWS individual ‘white’ noise (i), i.e. normally distributed, randomized values with zero mean and pre-defined variance. To assure, that these 1000 RWS on average represent the single signal from which they were generated, the added noise was designed such, that for each ‘year’ (j) along the RWS the average of all noise-values in the year (j) was zero. Thereby, if averaging all 1000 RWS to one single master chronology the exact original detrended Scots Pine RWS is obtained (see Fig 1). One would expect that any defined gradient for PP1 will originate from the added noise and responder groups thus will not enhance the strength of a common signal.

**Fig 2:**
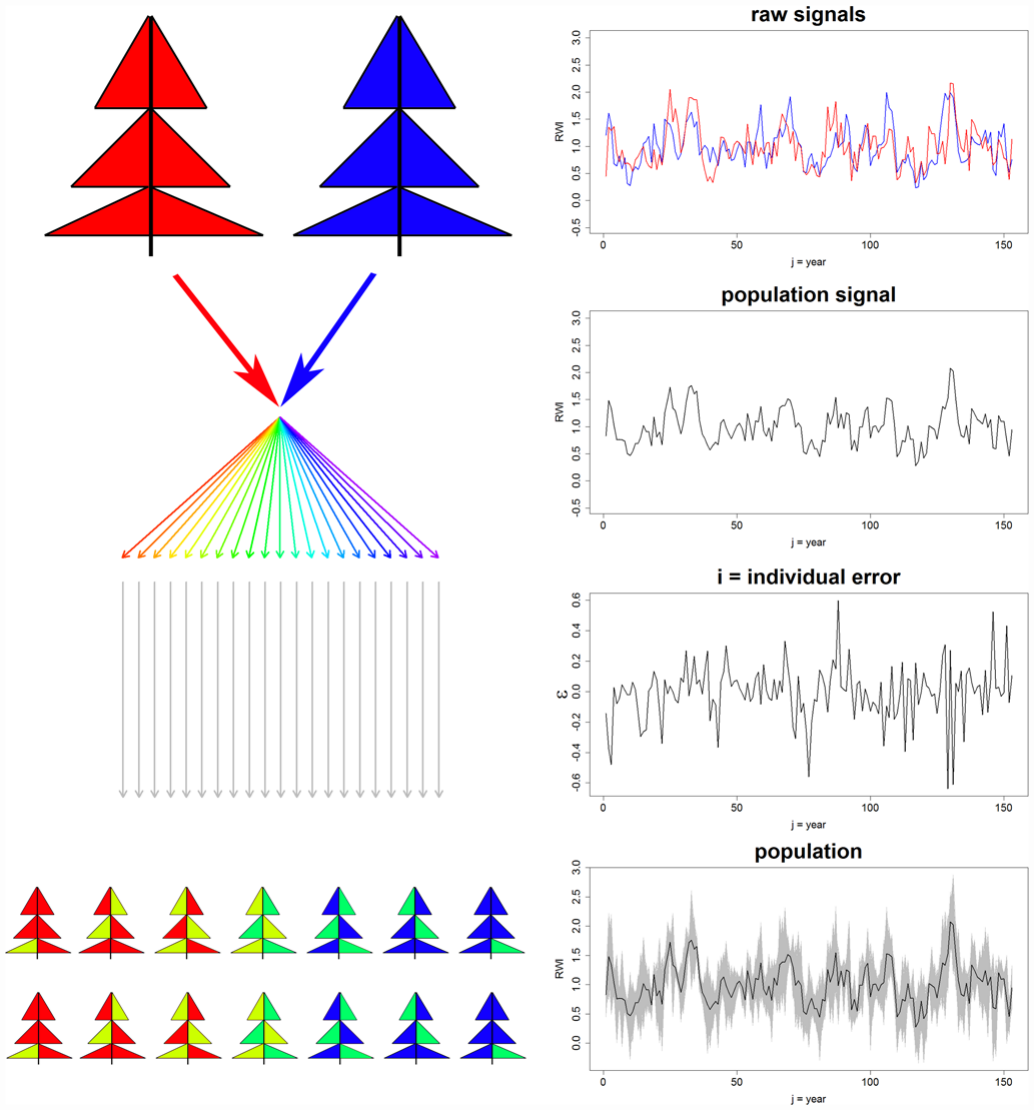
Generation of a heterogeneous pseudo-population. Two different signals (red and blue) are mixed along a linear gradient (rainbow coloured arrows) to obtain 1000 RWS with a gradual transition of underlying signals. As for PP1 individual-specific white noises are added.

**Fig 3:**
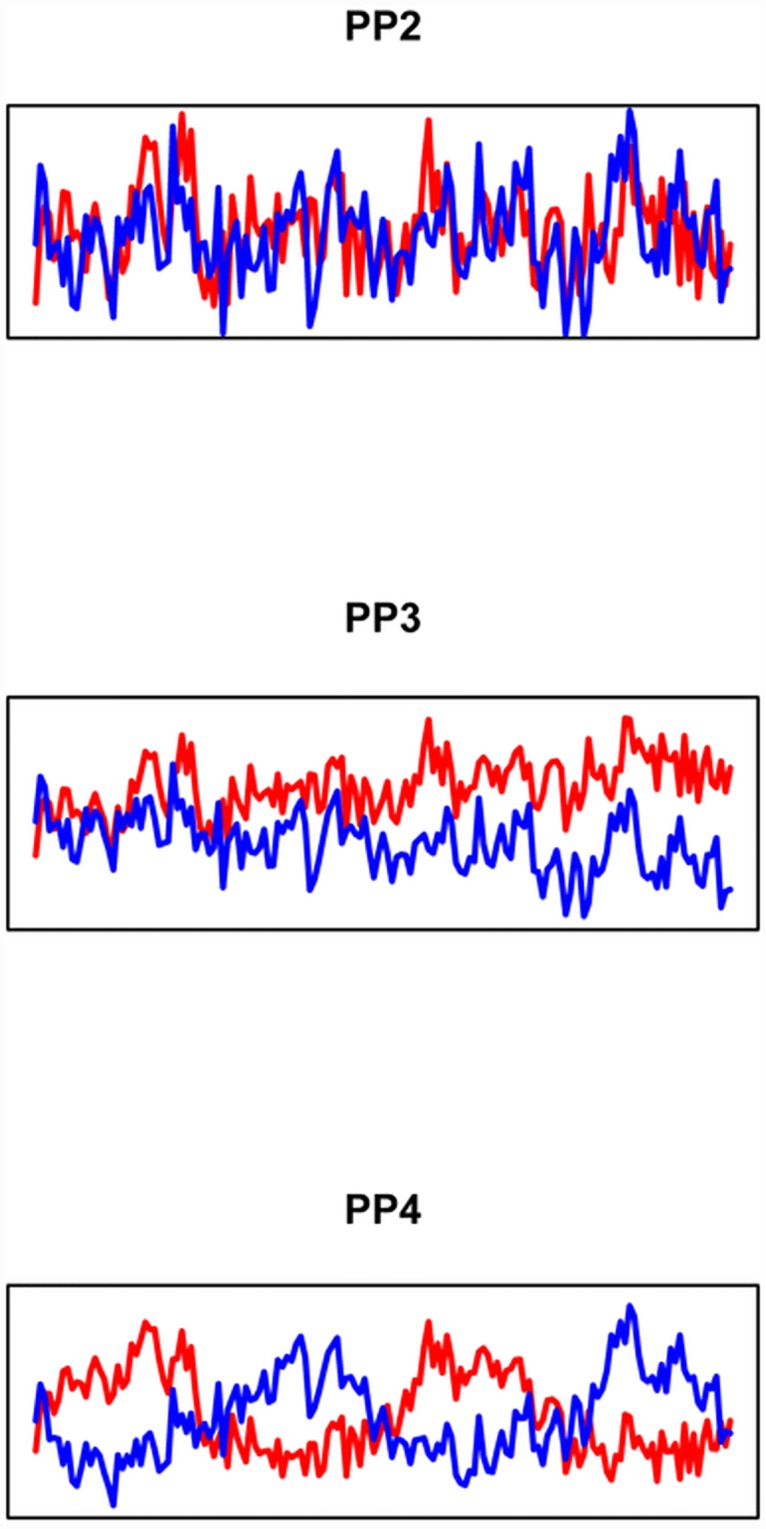
Long term signals of the pseudo-populations. The signals behind PPs 2–4 showing no (PP2) or differing long-term trends (PP3-4). To better visualize the differing long-term trends these were multiplied by factor 3.

For the heterogeneous PPs 2–4 we chose two different detrended Scots Pine RWS and mixed those along a gradient consisting of 1000 RWS. That is, the first out of 1000 RWS mainly consisted of one of the two signals (red in Fig 2), whereas the last consisted of the opposite signal (blue). Between these two extreme RWS a linear gradient was computed (rainbow coloured arrows in Fig 2). Following this approach, we assume to represent an artificial ecological gradient between two growth drivers, along which the RWS express a transitional change of growth signals. By this we are able to test whether the introduced approach meets applications I, II, and III (see introduction). Subsequently we added individual white noise to each of the RWS as for PP1. As PPs 2–4 consist of two different signals their population signal is exactly the average chronology of those two signals. The differences among PPs 2–4 were related to different long-term trends by which the signals were overlain. In PP2 no differing long-term trends occur (upper panel in Fig 3). For PP3 a positive (negative) slope was added to the red (blue) signal (mid panel, Fig 3), whereas we in PP4 added two subsequent sine (cosine) waves (Fig 3, lower panel). The Pearson correlation coefficients between the respective signals range from PP3: 0.15 to PP2: 0.38, glk of the two Scots Pine RWS was 0.57.

To represent more realistic conditions (realistic in terms of typical tree-ring datasets) we randomly subsampled each PP to obtain 200 RWS. For the representation of steep gradients with abrupt interruptions (i.e. representing a sample which did not fully resemble the existing gradient) we additionally subsampled each 100 RWS at each of the margins and in the center of PP4 resulting in PP4_X_. In terms of validation, a statistical approach should be able to sort RWS according to known gradients. Spearman rank correlation between known and detected gradient rankings should therefore be high (testing I and II, i.e. detection *of* and relation *to* ecological gradients). Furthermore, resulting responder chronologies should express higher signal correlations than the overall master chronology (testing III).

#### 2.1.2. Real world populations

To show PCGA performance using real world populations (see also section 2.5.), we re-sampled two classical tree-line sites in Alaska in 2012: Rock Creek Watershed (63°44’ N, 149°00’ W) in Denali National Park (DN), an elevational tree-line site, and Nutirwik Creek (67°56’ N, 149°45’ W) in the Brooks Range (BR) at the northern tree-line. Both stands were formed by mono-specific stands of White Spruce (*Picea glauca* M.), the main tree-line species in Alaska and north-western Canada. Per site we sampled 1) the highest elevation trees (hereafter referred to as DN:TL and BR:TL for Denali or Brooks Range tree-line, respectively) and 2) trees about 200 m (DN) and 50 m (BR) lower in elevation growing in more closed stands (hereafter referred to as DN:FO and BR:FO for Denali or Brooks Range forest, respectively). For all plots we sampled increment cores from all trees with DBH > 5 cm. All plots show clearly differing long-term growth trends, making them an ideal case study to exemplify our new approach. Permission to sample tree-cores in DN was granted by the United States National Park Service (Park-assigned Study or Activity #: DENA-00849, Park-assigned Permit #: DENA-2012-SCI-0019). For BR a casual use determination was granted by the Bureau of Land Management (Casual Use Determination #: 2920(AKFO3000)). *Picea glauca* is a frequently distributed tree species in North America and is therefore not considered a protected species.

We collected nearly exclusively penetrating cores, resulting in two RWS per tree which were averaged before further processing. Cores were measured using standard dendro-analytical techniques (conventional sanding, measurements with LINTAB and TSAPWin professional, cross-dating with COFECHA). Prior to analyses we chose a subsample of trees referring to a common age cohort at each site. For all sites but DN:TL, this age cohort was chosen from age 90 to 150 years. As the corresponding age cohort was only weakly represented at DN:TL (n = 16) we defined the respective age class from an age of 60 through 120 years. The resulting age cohorts comprised 52, 122, 58, and 47 trees for BR:TL, BR:FO, DN:TL, and DN:FO, respectively. Age cohorts were selected to minimize possible effects of long-term trends that relate to differences in age and establishment periods. The complete data was either 1) standardized using a horizontal mean or 2) detrended using the Modified Negative Exponential detrending procedure. Independent of the selected detrending both datasets showed comparable–though not identical–results. Here we focus on the horizontal mean standardization results to avoid possible differences in detrending treatments dependent upon the length of individual RWS. The effect of possible age-related growth trends on PCGA performance is estimated using squared Spearman correlation coefficients (r²) between detected PCGA gradients and tree age.

### 2.2. Four different approaches

To situate the new approach in the scientific context of individualistic growth response we compared two established methods (GR and TR) with PCGA focusing on their ability to detect known gradients within pseudo-populations (applications I and II). To further compare the ability of PCGA to define responder chronologies with more sophisticated methods than GR and TR, we compared PCGA with HCA (application III).

#### 2.2.1. The first approach: Growth ratio (GR)

The first approach is based on the calculation of growth ratios (GR). For this, the analyzed period was split into two windows of equal length and for each RWS the average value of the latter period was divided by the average of the first period. Thus, GR values above 1 indicate increasing values after the reference year (i.e. positive growth trend) whereas values below 1 indicate decreasing values (negative growth trend). Based on Wilcoxon rank sum test between values of the two periods it is possible to define significantly positive or negative RWS trends if p<0.05. Referring to established jargon we call these groups positive and negative responders, respectively. RWS with no significant change between the periods were called non-responders. Although this particular method has not been applied in the literature, it refines a method described by [21] with the additional determination of responder groups based on p-values. By this it becomes possible not only to separate positive from negative responders but also both from non-responders.

#### 2.2.2. The second approach: Growth trend (TR)

In the second approach, we calculated linear regressions on RWS over the whole period. The slopes of these regressions can be seen as individual growth trends (TR). Here, positive (negative) responders were defined as RWS which slope was significantly higher (lower) than zero, whereas non-responders had non-significant slopes. This approach largely reflects the methods used in [9, 12, 14].

#### 2.2.3. The third approach: Hierarchical Cluster Analysis (HCA)

As the approaches GR and TR have certain drawbacks (see sections 1 and 4) we also considered to compare PCGA with Hierarchical Cluster Analysis (HCA). HCA has for instance been used by [20] to define responder chronologies. While [20] used Euclidean distances among 17 climate correlations of each tree as dissimilarity measure, we here use Euclidean distances among RWS. This was necessary to make HCA applicable to the PPs, for which we only were able to generate a maximum meaningful number of two climate signals as being artificial data. As in [20], HCA was performed on the dissimilarity matrix using Ward’s minimum variance method. The number of selected clusters was based on a careful visual interpretation of the branch lengths.

#### 2.2.4. The new approach: Principal Component Gradient Analysis (PCGA)

The proposed new approach was based on Principal Component Analysis (PCA). For the PCA we used scaled RWS as variables, i.e. each RWS was divided by its standard deviation which is a common procedure prior to PCA to achieve equal variance of all variables [24]. As PCA needs finite values in each of the observations (here: years) but RWS data due to the differing ages of trees will show an increasing number of missing values back in time, PCA was performed on the common interval, i.e. a period for which all considered RWS have finite values. Relative importance values of the first two principal components (PC) were calculated to express their importance with respect to the overall population variance.

PCA loadings represent the correlation of variables (in this case RWS) with the respective PCs, with positive (negative) loadings indicating that the respective variable is positively (negatively) correlated with the PC. If the first two axes explain a fair amount of overall variance (e.g. > 50%), variables with similar loadings on those axes will be highly correlated with each other, whereas such with dissimilar loadings will correlate less or–depending on the dissimilarity of loadings and the importance of axes–even negatively [25]. Therefore, RWS with similar loadings will show similar temporal patterns, indicating the response to a common driver. It follows, that the higher the deviation among loadings of two RWS, the less they will represent a common signal. Consequently, sorting RWS according to the arrangement of arrows theoretically allows for defining population gradients, which may be caused by population-internal or external variability (for examples of causes we refer to application I in section 1). The typical PCA biplot makes use of this circumstance by plotting loadings of the first two PCs as arrows, thereby visualizing variables similarity and in our context which RWS react on similar growth drivers.

To define population gradients (I and II), PCGA transforms the loadings of the first two PCs into polar coordinates. By this PCGA for each RWS achieves its angle (in proportions of pi) *around* and its distance *to* the origin of the rotation matrix (PC1 = 0, PC2 = 0). Theoretically, arrows with same directions and therefore equal angles respond to a similar signal. However depending on the equality of their distance to the origin, they will be more (low distance) or less (high distance) obscured by other signals, i.e. noise with respect to the indicated signal. This information is considered in the calculation of responder chronologies (see below). Concluding, PCGA uses the achieved angles to define a continuous gradient along dominant signals. As this defines a population gradient using PCA we call our approach Principal Component Gradient Analysis (PCGA).

To achieve responder chronologies (III), PCGA determines distinct population borders which separate RWS representing different signals from each other. For this, PCGA initially inspects the angle distances for significant breaks. I.e. whenever the distance of two neighbouring angles exceeds 4 standard deviations of all distances, this relatively abrupt change is defined as a border. Visually this border can be seen in PCA biplots if groups of arrows close by each other are separated by comparably large gaps (a respective example is PP 4_x_). However, if a gradient is sampled at a high frequency, such abrupt changes may not occur (e.g. PPs 2–4 when sampled completely). Therefore, PCGA in a second step determines (additional) borders based on Pearson correlations. For this, a ‘seed’ chronology is built at the margins of the distinct sub-populations, or—if none were identified–at the margins of the overall population. The number of RWS contributing to the ‘seed’ chronology here was chosen to be 10 percent of the overall population size, to achieve statistically representative sample sizes for the seed chronologies. For each tree contributing to the seed chronology its correlation to the seed chronology is calculated. The resulting seed chronology correlations are used as criterion for the inclusion of further RWS along the population gradient. I.e. if the moving average (with window size also being 10 percent of population size) of RWS correlations along the population gradient differs by more than 4 standard deviations from the seed chronology correlations, all RWS within the respective window along the gradient are defined as belonging to a new subpopulation. We chose to use moving window correlation averages to be conservative in our determination of responder chronologies. That is, single RWS along the gradient may express low correlations with an adjacent seed chronology if they are substantially obscured by noise (indicated by a comparably low distance to the origin of the rotation matrix), although subsequent RWS may still be correlated well with the seed chronology. Thus, borders identified at those particular RWS would not be correct. For each new defined population boundary, the procedure is repeated until the complete gradient has been analysed. Minimum sample size of resulting responder chronologies is again 10 percent of the overall population size, wherefore a maximum number of 10 responder chronologies cannot be surpassed. One may consider increasing the 10 percent threshold for small populations to retain statistically representative seed chronologies. To account for the differing noises of RWS along the population gradient, distances to the origin of the rotation matrix of each RWS are used as a factor to weigh the RWS’ contributions to the responder chronology. I.e. if RWS have comparably low distances to the origin, they will only weakly contribute to the respective responder chronology. As a measure of growth divergence, we here report the minimum correlation and glk among responder chronologies.

To quantify the extent to which PCGA gradients allow for enhancing the strength of a common signal we used the mean inter-series correlation (rbar) [e.g. 4]. In dendrochronology rbar is frequently used in the expressed population signal to quantify how well a finite sample represents a theoretically infinite sample [4]. In our approach we performed Monte Carlo simulations with 100 permutations (i) to estimate an average expected rbar for each sample size N between 2 and e.g. 50

$$\bar{Exrbar_{N}}=\Sigma_{i=1}^{100}\left(\Sigma_{j=1}^{N}\Sigma_{k=j+1}^{N}cor\left(RWS_{j},RWS_{k}\right)\right)\cdot \frac{1}{100}\;\;;N\;:=2\ldots 50$$ (1)

Next, we calculated the two gradient rbar (*grbar*_*N*_) sequences obtained from RWS along the PCGA gradient at both margins with successively increasing sample size. I.e. first *grbar*_2_ of the two ‘reddest’ (‘bluest’) RWS was calculated and then we included one RWS after another along the gradient (*grbar*_3,_ *grbar*_4_ … *grbar*_50_). The relative rbar expressed as percentage $Rrbar_{N}=(grbar_{N}/\bar{Exrbar_{N}})\;\cdot \;100\%$ allows for quantifying the enhancement of the population signal above an average random subsample for each N. It seems meaningful to calculate *RrbarN* for each defined responder chronology to quantify their respective signal strength enhancement in comparison to a random sub-population chronology with equal sample size. We decided to use rbar instead of the expressed population signal (EPS [4]) as EPS strongly is affected by sample size, this obscuring the factual increase of rbar for responder chronologies with large sample sizes. This measure we call the relative responder chronology signal strength (RRCSS).

### 2.3. Evaluating the approaches

#### 2.3.1. Gradient detection

To compare the performance of GR, TR and PCGA with respect to gradient detection we applied each approach to pseudo-populations (see 2.1.1.). As existing population gradients were known, PP allowed us to correlate the ranking of each approach with known gradients using Spearman’s rank correlation. The higher Spearman’s rank correlation between estimated and known gradients, the better the performance with respect to applications I and II.

#### 2.3.2. Potential effects on climate transfer functions

With respect to the performance of PCGA responder chronology definition we quantified the enhancement of rbar using RRCSS as described in 2.2.3 (III). To highlight potential effects of responder chronology definition on climate transfer functions (i.e. models used to predict climate parameters from RWS), we computed individual correlations for each RWS. For pseudo-populations we correlated each RWS with the signal(s) behind the PP (see section 2.1.1.). In addition, we computed the corresponding signal correlations with the overall master chronology (i.e. including all RWS), and the PCGA and HCA responder chronologies. The resulting correlations are described and expressed as average Δr² between the population master and the responder chronologies (application III). Please note that to allow for comparability with HCA which sometimes detected groups with sample size lower than the PCGA minimum sample size (10 percent of population size), we for BR:FO and DN:FO show both the standard PCGA performance and results from analyses where we overrode the minimum sample size criterion to allow for equally small sample sizes. To visually compare all computed correlations we plotted the results as in [5], however with the extension of colouring single tree correlations according to their PCGA ranking.

### 2.4. Real world application of PCGA

To further examine the performance of PCGA we applied it to the Alaskan data-sets (see 2.1.2.). PCGA responder groups were defined and signal enhancement was quantified using RRCSS. Climate correlation analyses were performed against gridded monthly temperature averages and monthly precipitation sums from 1901 to 2009. Climate data (CRU TS 3.1) were downscaled by SNAP (Scenarios Network for Alaska and Arctic Planning 2013) via the delta method [26, 27] and interpolated to a 2 by 2 km spatial resolution with the Parameter-Elevation Regressions on Independent Slopes Model (PRISM). From temperature and precipitation we further calculated the Standardized Precipitation Evaporation Index (SPEI) integrated over 3 and 6 months as measures for plant water availability [28].

As the ‘true’ gradient for real-world data is not known, we were not able to test the correct identification of gradients (I). As an example for application II and to test, whether detected population gradients just reflect age related growth differences, we correlated population gradients against tree age. As for the pseudo-population signal correlations, we performed individual RWS as well as responder and master chronology climate correlations (III).

All analyses were programmed within the ‘R’ environment (version 3.2.0) using packages ‘dplR’ [29] and ‘SPEI’. Code relevant for PCGA calculations mentioned under 2.2.3 is shown in the supplementary, S1 Rcode. A respective ‘R’-package is under preparation.

## PCGA Performance

### 3.1. Pseudo-population based evaluations

**Fig 4:**
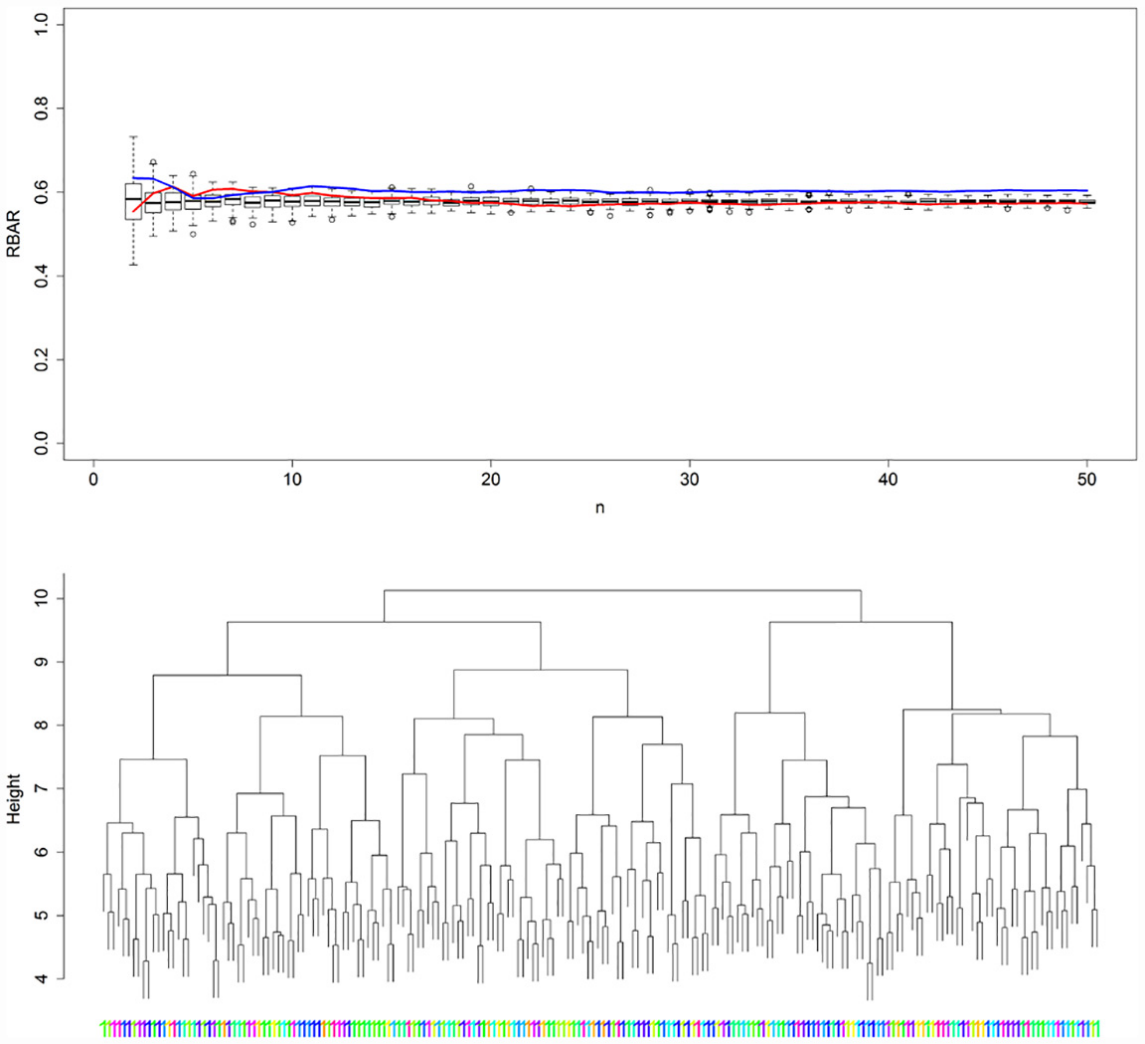
Evaluation plots for PP1. Upper panel: *grbar*_*N*_ plotted against $\bar{Exrbar_{N}}$ along the population gradient for both population margins (red and blue lines). The blue line suggests a slightly higher *grbar*_*N*_ along the gradient. Lower panel: HCA dendrogram suggests defining only one responder chronology, however with weak indications of sub-populations, probably caused by noise similarity. HCA labels indicate to which cluster each RWS belongs (here each belonging to cluster 1), while their colours relate to their position within the known gradient. As no gradient exists within PP1, the colours are mixed randomly.

For the homogeneous PP1, PCGA correctly determined only one responder chronology, i.e. the population master chronology. However, when evaluating *grbar*_*N*_ along the population gradient, it seems to be slightly enhanced for one of the population margins (Fig 4, upper panel), this reflecting an above average similarity of the added noise at this edge of the population. HCA dendrogram inspection also suggested defining only one chronology (Fig 4, lower panel) despite that the upper branch lengths, gave a weak indication for potential groups, probably indicating the similarity of added noise within possible groups. For detailed statistics we refer to Table 1.

**Table 1:** Detailed statistics of the PP based evaluations.

PP	r²		Gradient r²			N (chrons)		cor	glk	RRCSS [%]	Δ r²	
	PC1	PC2	GR	TR	PCGA	PCGA	HCA				PCGA	HCA
1	0.58	0.01	---	---	---	1	1	1	1	100	0	0/0
2	0.44	0.05	0.01	0.00	0.90	3	2	0.74	0.76	110/108/108	0.15	0.12
3	0.23	0.08	0.92	0.92	0.94	4	4	0.17	0.66	109–155	0.39	0.40
4	0.44	0.12	0.00	0.74	0.98	2	3	0.58	0.72	126/117	0.16	0.18
4_x_	0.40	0.17	0.00	0.77	0.87	3^1^	3^1^	0.13	0.65	157/122/156	0.19	0.19

PC1/PC2 r² refers to the amount of variance explained by PC1 and PC2. Gradient r² shows the amount of true gradient variance explained by the gradient obtained by GR, TR, and PCGA, respectively. Columns 3–6 show 3: the number of defined PCGA and HCA responder groups, 4: the minimum correlation and glk between PCGA responder chronologies, 5: the corresponding RRCSS (see section 2.2.3.) and 6: the average signal strength enhancement for PCGA and HCA chronologies (see section 2.3.2.). Positive values in column 6 indicate that the responder chronologies explain more variance of the underlying signal than the population master.

^1^both PCGA and HCA perfectly determined the three distinct responder groups

**Fig 5:**
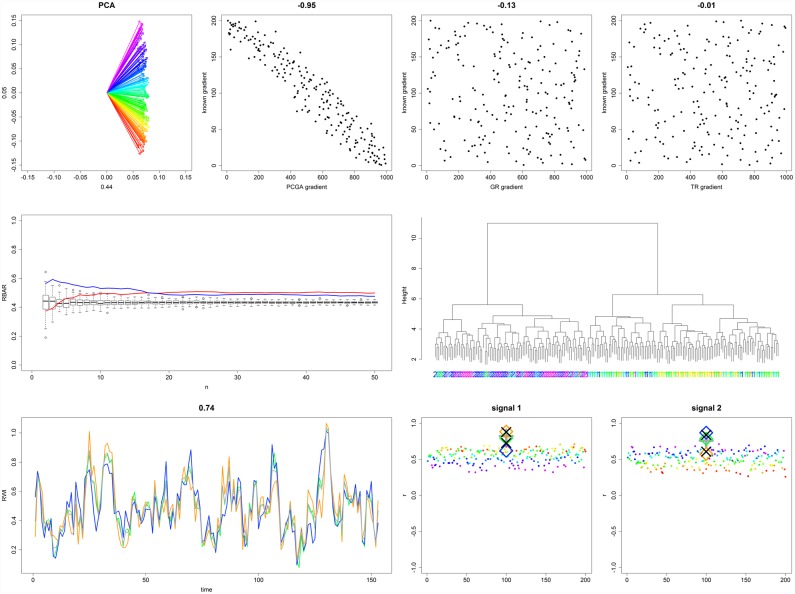
Evaluation plots for PP 2. (for a higher resolved image we refer to supplement S1 Fig). Upper left panel: Loadings of the PCA over RWS coloured according to the PCGA gradient. Axis labels refer to PC relative importance. Three upper right panels: Detected gradients plotted against known gradients for PCGA, GR, and TR, respectively. Headers of these panels reflect the correlation coefficient between detected and true gradient. Mid left: *grbar*_*N*_ plotted against $\bar{Exrbar_{N}}$ along the population gradient for both population margins (red and blue lines). Both *grbar*_*N*_ strongly suggest a population gradient as being well above $\bar{Exrbar_{N}}$. Mid right: HCA dendrogram suggested defining two responder chronologies which mostly correctly split RWS along the known gradient. Lower left: PCGA responder chronologies (coloured curves) show a minimum correlation of 0.74 among each other. Two lower right panels: RWS signal correlations (small dots coloured according to PCGA gradient) show a clear relationship with PCGA gradient. PCGA (coloured ‘◊’) and HCA (black ‘X’) responder chronologies show equal signal correlations of which the marginal responder chronologies express higher correlations than the population master (grey ‘+’).

**Fig 6:**
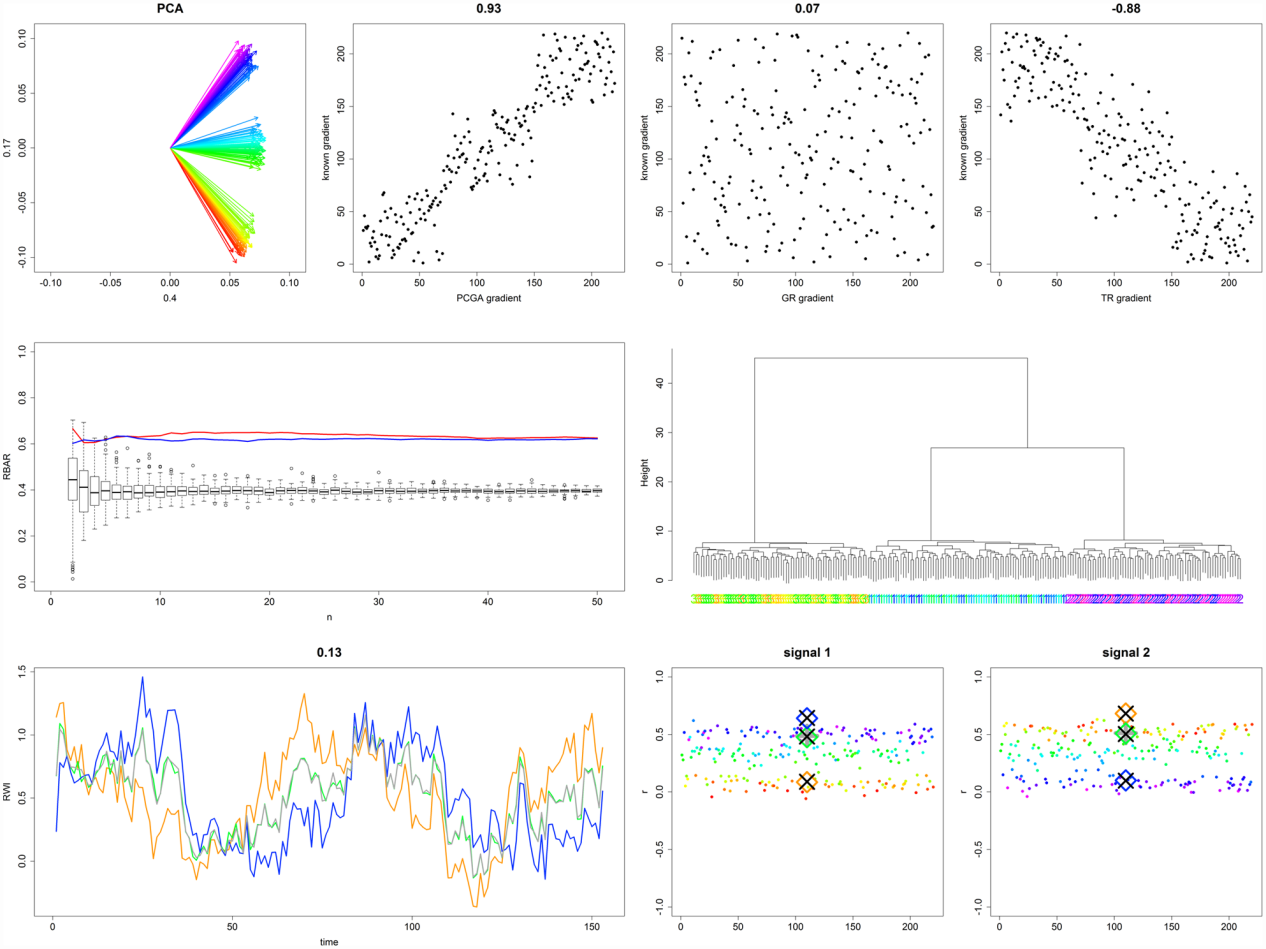
Evaluation plots for PP 4_x_. For detailed explanations we refer to the caption of Fig 5.

Regarding the heterogeneous PP 2–4, mentioned statistics are presented in Table 1. Here, we focus on PPs 2 and 4_x_ (Figs 5 and 6) but refer to supplement S1–S4 Figs for high resolution images of all heterogeneous PPs. For PP 2 GR and TR failed to detect the population inherent gradient, expressed by gradient r² close to zero. In contrast, PCGA correctly identified the population gradient with 90 percent of explained variance. PCGA determined 3 responder chronologies, which expressed RRCSS around 109 reflected in signal explained variance being on average 15 percent higher if compared to the population master. HCA dendrogram suggested defining 2 responder chronologies, which expressed slightly lower signal strength enhancement.

For PP 4_x_ GR failed to detect the gradient, while TR performed comparably well but was superseded by PCGA (gradient r² being 0.00, 0.77, and 0.87 respectively). PCGA and HCA both correctly determined the three sub-populations and thus showed exactly the same signal strength enhancement. RRCSS ranged from 122 for the central chronology up to 157 for the marginal responder chronologies. Summarizing, from our PP evaluations PCGA seems to be capable of applications I-III mentioned in section 1.

### 3.2. Real world data

**Fig 7:**
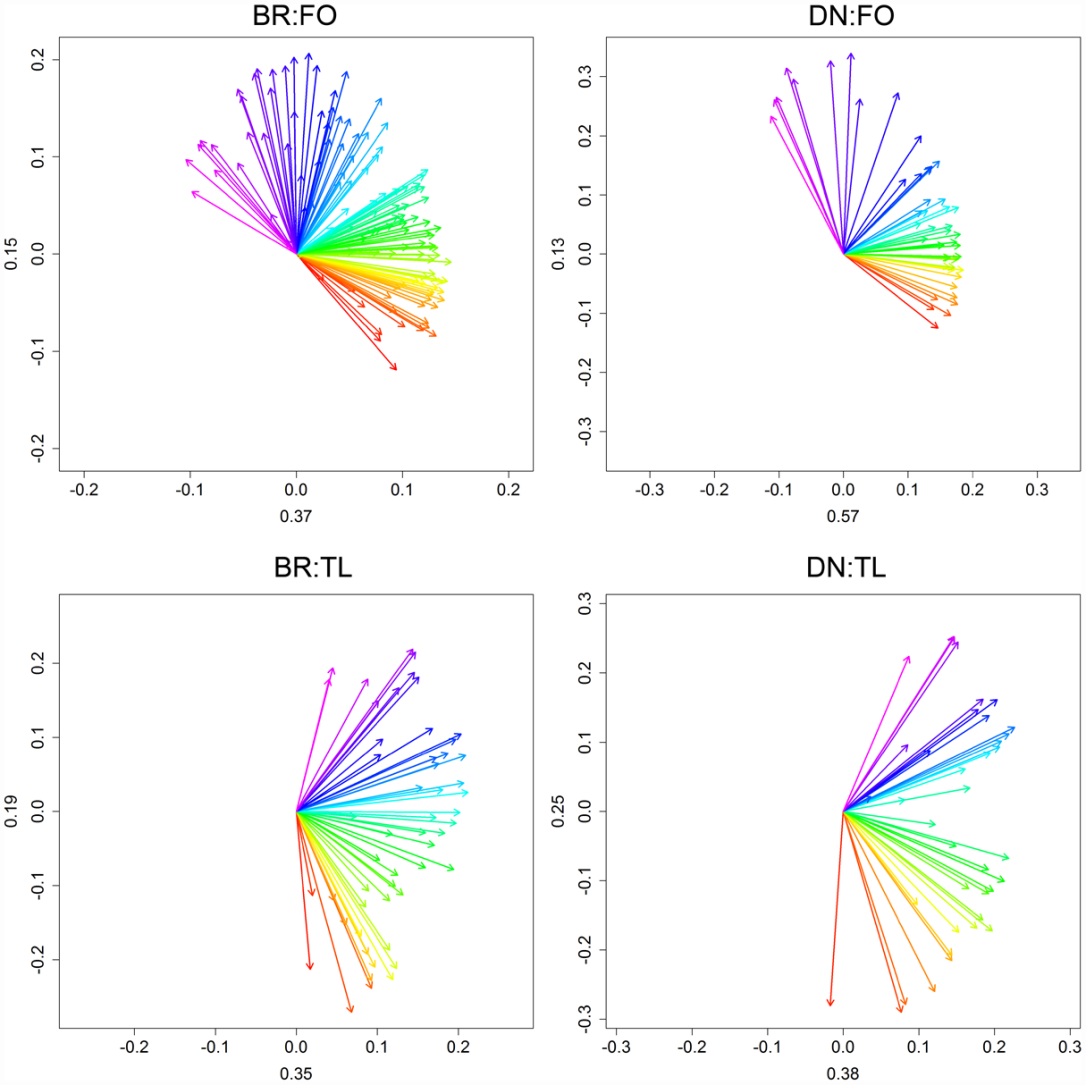
PCGA plots for the Alaskan sites. PCA loadings coloured according to PCGA gradients for the four Alaskan sites. At each site PCA loadings suggest ecological gradients supported by comparably high explained variances on the first two PCs (values are given for each PC).

**Fig 8:**
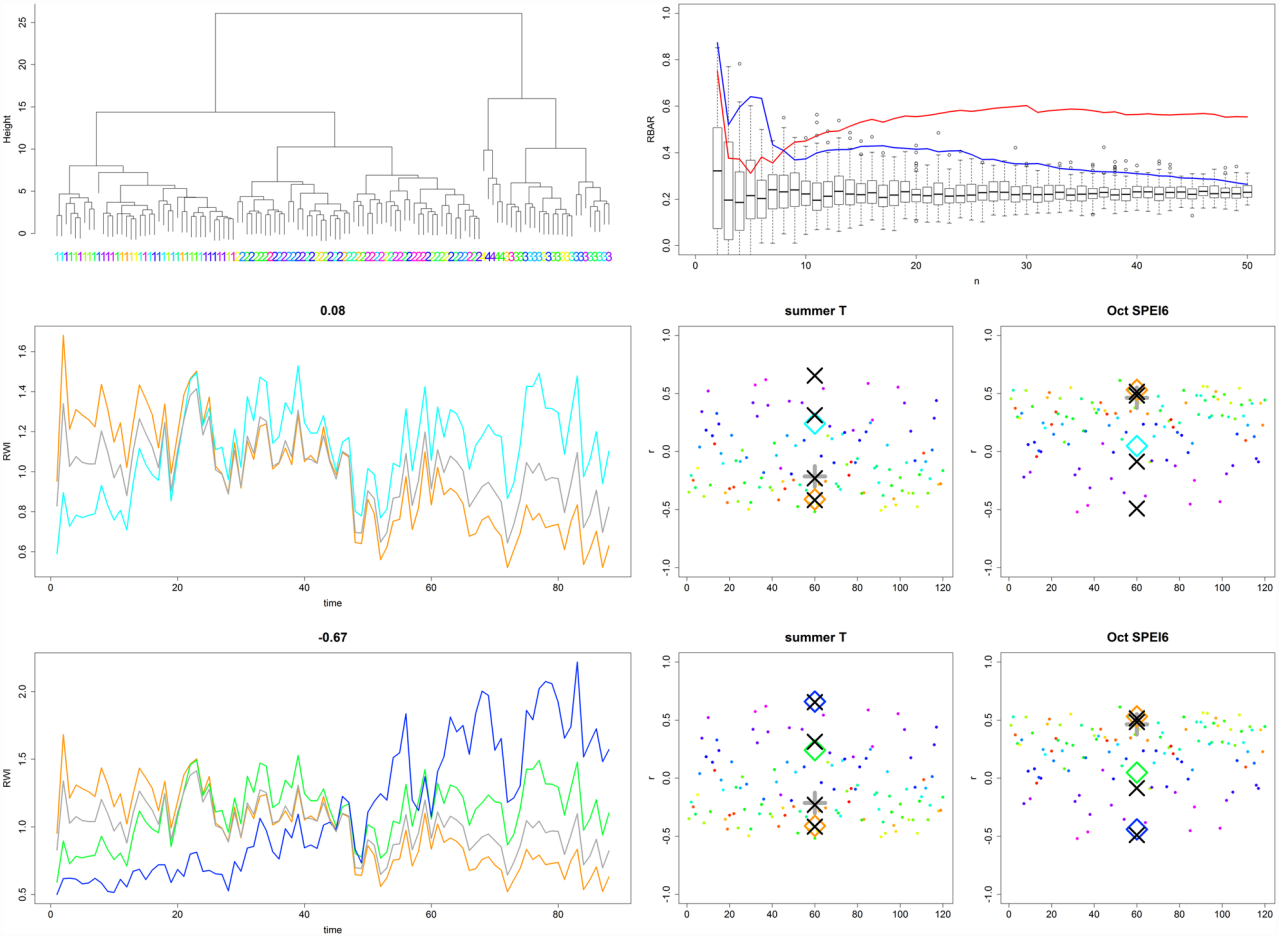
Evaluations plots for BR:FO. Upper left: HCA dendrogram suggests selection of four responder chronologies. Note, that cluster four only consists of five specimens. Upper right: *grbar*_*N*_ clearly indicates signal strength enhancement at the margins of the population. Mid left: PCGA extreme responder chronologies show weak correlations with each other. Mid right: PCGA and HCA responder chronology signal correlations are comparably strong: see also Table 2. Lower left: Same as mid-left but here for the analyses where the minimum sample size was adjusted to match HCA minimum sample size. Lower right: Same as mid right but here for the analyses where the minimum sample size was adjusted to match HCA minimum sample size.

Statistics of the real world data evaluations are shown in Table 2, Figs 7 and 8 and S5–S8 Figs. Here we focus on the case of BR:FO. For all four datasets, PCGA explained at least 50 percent of variance along the first two PCA axes (Fig 7). Resulting *grbar*_*N*_ indicated a significant gain of signal strength for each site, supported by RRCSS > 120 and positive Δ r² of at least 10 percent if compared to the population master.

**Table 2:** Evaluation statistics for the Alaskan data.

Site	PC1	PC2	Age r²	N (chrons)		cor	glk	RRCSS [%]	Δ r²	
				PCGA	HCA				PCGA	HCA
BR:FO	0.36	0.15	0.00	2	4	0.08	0.88	214/121	+0.10	+0.20
BR:FO^1^	0.36	0.15	0.00	4	4	-0.67	0.74	213/144/173	+0.22	+0.20
BR:TL	0.34	0.19	0.02	3	3	-0.32	0.85	150/152/178	+0.20	+0.16
DN:FO	0.57	0.13	0.13	4	5	-0.33	0.78	133–202	+0.12	+0.13
DN:FO^1^	0.57	0.13	0.13	5	5	-0.66	0.62	122–200	+0.13	+0.13
DN:TL	0.43	0.16	0.06	4	4	-0.15	0.79	123–239	+0.10	+0.08

Same statistics as in Table 1 but for the Alaskan data and with r² of age on the gradient. Number of trees are 122, 52, 47, and 58 for BR:FO, BR:TL, DN:FO, and DN:TL, respectively.

^1^ for these populations the minimum sample size of responder chronologies was adapted to match HCA responder chronology sample size,

When compared to HCA in the context of application III, PCGA responder chronologies performed comparably well however with some specifications needed for the forest plots (see S5 and S7 Figs). For BR:FO this is to be explained by the fact, that BR:FO seems to contain a sub-population of only 5 specimens (cluster 4 in HCA). In contrast to HCA, PCGA responder chronologies have a minimum sample size of 10 percent of the total population size (i.e. 12 for BR:FO), wherefore the latter is not able to resemble this specific responder chronology. If allowing for comparably low sample sizes in PCGA, the respective responder chronology is recognized and signal correlations perform comparably well (Fig 8 lower panels vs. mid panels, Table 2). The same is to be observed for DN:FO for which HCA determined two clusters consisting of 4 and 5 specimens respectively (S7 Fig).

## Discussion

### 4.1. Comparing PCGA to other approaches

In our comparative evaluation (section 3.1.), we were able to show that PCGA in general was able to detect population gradients, thereby meeting I and II (see introduction). In contrast, GR and TR failed for particular PP. This is to be explained by the approach specific designs which rely on linear growth trends over time. PP 2 and 4 were particularly designed to express no linear growth trend over time (Fig 3). As PCA loadings reflect the inter-correlations of variables [25] which will identify contrasting RWS of any sort, PCGA allowed for successful detection of these gradients. For the Alaskan data, GR and TR were also able to define comparable–though slightly differing–responder groups if focusing on specific periods (not shown) as also shown by earlier investigations [7, 30]. However, for other periods or data which express specific behaviour, GR and TR–in contrast to PCGA–will fail as indicated by our comparative evaluation.

With respect to application III, PCGA and HCA performed equally well, expressed by similar signal strength enhancement in comparison to the population master. However, for BR:FO and DN:FO, PCGA was only able to compete with HCA, if the minimum sample size of responder chronologies was reduced below the threshold of 10 percent of the overall population sample size. The reasons for this is, that HCA has no restrictions with respect to minimum sample size, whereas we believe that such a threshold is meaningful to end up with statistically representative responder chronologies. If overriding this threshold, PCGA detected equally well performing responder chronologies. In terms of tree-ring based climate reconstructions (the main purpose of signal strength enhancement), it is rather unlikely that responder chronologies with such low sample sizes (4–5) will allow for reliable climate reconstructions. An option to deal with such underrepresented responder chronologies may be to define the causes for their deviating growth trends. Here PCGA theoretically may be of help by correlating the population gradient against internal and external variables (see II in section 1). If causes were identified, additional tree-cores may be obtained specifically aiming at trees which represent comparable conditions of the respective sub-population (e.g. extremely moist or dry sites).

Although HCA performed equally well with respect to responder chronology definition we believe that PCGA is advantageous. Namely, as soon as it comes to an explanation of HCA sub-populations, the obtained clusters will only allow for a category based exploration, for instance using ANOVA or Kruskal Wallis test and their related post-hoc tests. The same (categorical groups) holds for the PCA approach suggested by [23] as they defined groups according to high loadings on the respective principal components. In contrast, PCGA allows for analyzing gradients along a continuum (as GR and TR, which have their own disadvantages as described above) and therefore at a much higher resolution. Hence we conclude that PCGA combines the advantages of other approaches in one single approach with no significant drawbacks.

### 4.2. Potential applications and limitations of PCGA

Our analyses clearly demonstrate the potential benefit of enhancing climate signals using PCGA. As responder group definition is based on explorative tools (PCA) which are not directly related to climate correlation analyses, this approach cannot be considered ‘cherry picking’ [6]. In the mentioned context ‘cherry picking’ would be to choose RWS which show highest correlations with environmental signals for reconstruction of those. In contrast, PCGA defines statistically supported responder groups *prior to* climate correlation analyses and reconstructions. We therefore consider PCGA with subsequent responder group definition to be an objective means that allows for enhancing the strength of climate reconstructions in a dendroclimatological context.

Beyond the enhancement of common signals, PCGA allows for exploring occurring population gradients. If analyzing environmental data along PCGA population gradients, the reasons for those gradients may be elucidated. In our PP the gradients were related to the gradual transition of two signals (see 2.1.1.) which always were correctly determined (high gradient r² in Table 1). In the Alaskan examples we correlated age against the population gradient so see whether the different growth trends were explainable by age related growth differences. Of course any other tree-based metadata such as competition indices, tree size, micro-site topography, soil properties, disturbance history, etc. could be used in this way to possibly explain detected gradients. By this, it may also become possible to explain differing climate correlations, which from a dendroecological point of view is of even higher interest than increasing the strengths of environmental signals. From the arrangement of colours in our climate correlation plots (lower right panels in Figs 5, 6 and 8) it becomes clear that climate correlation coefficients are related to PCGA gradients.

As an example, responder groups from BR:FO expressed differing responder chronology correlations with summer temperature and summer SPEI (see Fig 8). Although not being able to explain these differences with the available metadata, these correlations may indicate drought stress for the negative responder group which could be related to differing micro-site soil properties. For the Denali we also found contrasting summer temperature as well as current year winter SPEI correlations (S7 and S8 Figs). Although SPEI correlations were slightly weaker as for the Brooks Range this may again point towards summer drought stress (although not shown summer SPEI also showed significant correlations) and in addition winter snow cover–effects that theoretically could be explained by micro-topographical variations. By re-sampling sites for respective metadata (soil properties and micro-topography) these hypotheses could be tested for by correlating PCGA gradients against these data.

We want to stress that PCGA responder groups supported earlier findings [7], namely that RWS responder groups reacted differently to a shift in the Pacific Decadal Oscillation around 1970 which resulted in warmer and drier conditions in interior Alaska [31, 32]. This possible connection is supported by the summer SPEI correlations found for BR:FO and highlights another potential application of PCGA, namely event detection. Our analyses suggest that PCGA is able to detect different growth responses to particular disturbances (climate shifts, storms, fires, calamities, management, etc.) after which specific trees may benefit from decreased competition whereas others may suffer from the stressors which were responsible for the disturbance.

In contrast to the shown examples, there may be cases where growth divergence emerges abruptly and/or occurs with a certain delay to its cause(s). For instance groundwater withdrawal after a dike establishment may lead to successively increasing drought stress of trees growing on well drained soils, whereas trees from poorly drained soils hardly will be affected. As groundwater–depending on climate and geological conditions–reacts slowly (several years to decades), growth divergence may emerge much later than the actual event. In such case identifying the causes of growth divergence may be difficult. A possibility to cope with this is moving window PCGA. That is, PCGA is performed for several time-windows of equal size (e.g. 30 years) along time to separate periods in which growth divergence was absent from periods with existing growth divergence. In the mentioned example one would expect no or only weak population gradients before the dike establishment, whereas population gradients successively should become stronger after the event. Careful inspection of emerging population gradients over time then may allow for identifying the cause of abrupt growth divergence. Respective results may be supported by moving window climate correlations of the detected responder groups which in the mentioned example would indicate increasing drought stress for trees growing on well-drained soils after the dike establishment.

Although PCGA appears powerful we want to stress the following important points. As with all statistical explorative tools, results should be interpreted carefully and it should be assured to take into account all potential causes for detected population gradients. With respect to this, we pre-selected age cohorts to lower the probability of age related growth differences. While this approach was successful for three of the four real-world sites, minor effects (r² = 0.13) of age on the PCGA gradient were observed for DN:FO. It is important to note that if PCA loadings express strong variations with respect to distance to the origin of the rotation matrix, this is a strong indication for substantial additional noise in the data. This will however also be reflected in low explained variances of the first two principal components. In such case, detected gradients and defined responder chronologies should be interpreted and used with particular caution. Here, comparison with HCA–which however also will be affected by substantial noise expressed by short branches in the dendrogram–may support PCGA responder chronology definition.

In the context of age related effects, trend removal within RWS (detrending)–a standard procedure in dendro-climatology [1]–needs to be mentioned. We also applied PCGA to negative exponential detrended data with slightly differing but in general similar results. I.e. also there PCGA clearly detected responder groups and allowed for signal strength enhancement (not shown). However, other detrending techniques such as polynomial functions (‘smoothing spline’) which tend to remove also periodical trends delivered completely differing results, as the decadal growth trends which cause the observed dissimilarities among RWS were removed (not shown). Detrending is a highly debated topic in dendrochronology and we are aware of that to reconstruct climate without effects from age or size related RWS trends one should make sure to account for these trends correctly. As the main aim of this methodological paper is to present the performance of PCGA not only in dendroclimatological but also dendroecological context, we presented the horizontal mean standardized data. This we did because differing absolute growth trends–whatever may be their cause–very well are of high interest to dendroecologists. Concluding, when considering using PCGA, detrending will be a case-dependent and carefully to choose decision.

A potentially limiting factor for PCGA is related to the sample size of responder groups. As shown for the real world forest plots, responder groups equal to those from HCA were only detected when lowering the minimum sample size threshold for definition of groups. While it in the context of sub-population detection seems justified to define responder groups with low sample size, it in terms of climate transfer functions is less likely that responder chronologies with small sample sizes will deliver stable and thereby reliable climate reconstructions. Therefore we want to stress the importance that one should carefully decide whether to work with the defined responder chronologies and propose re-sampling of under-represented groups if aiming at climate reconstructions.

## Conclusions

In this methodological study we were able to show that a new approach–the Principal Component Gradient Analysis (PCGA)–performed well and in particular situations (PPs 2, 4, and 4_x_) better than other approaches (GR and TR) with respect to population gradient detection and comparably well in comparison to clustering techniques (HCA) in the context of responder chronology definition. In contrast to other relevant existing explorative tools [20, 23] PCGA allows for detection of continuous gradients. Our analyses indicate that PCGA for the shown scenarios is able to correctly identify population gradients and to enhance the strength of tree-ring based climate reconstructions. In metaphoric words: PCGA correctly tunes the voices within a choir. We therefore conclude that PCGA may be of particular interest both for dendro-ecologists and dendro-climatologists but potentially also for scientists beyond dendrochronology working on population based data of comparable structure.

## Supporting Information

S1 FigEvaluation plots for PP2.Upper left panel: Loadings of the PCA over RWS coloured according to the PCGA gradient. Axis labels refer to PC relative importance. Three upper right panels: Detected gradients plotted against known gradients for PCGA, GR, and TR, respectively. Headers of these panels reflect the correlation coefficient between detected and true gradient. Mid left: *grbar*_*N*_ plotted against $\bar{Exrbar_{N}}$ along the population gradient for both population margins (red and blue lines). Both *grbar*_*N*_ strongly suggest a population gradient as being well above $\bar{Exrbar_{N}}$. Mid right: HCA dendrogram suggested defining two responder chronologies which mostly correctly split RWS along the known gradient. Lower left: PCGA responder chronologies (colored curves) show a minimum correlation of 0.74 among each other. Two lower right panels: RWS to signal correlations (small dots coloured according to PCGA gradient) show a clear relationship with PCGA gradient. PCGA (coloured ‘◊’) and HCA (black ‘X’) responder chronologies show equal signal correlations of which the marginal responder chronologies express higher correlations than the population master (grey ‘+’).(TIF)

S2 Fig Evaluation plots for PP 3. For detailed explanations we refer to the caption of S1 Fig.
(TIF)

S3 Fig Evaluation plots for PP 4. For detailed explanations we refer to the caption of S1 Fig.
(TIF)

S4 Fig Evaluation plots for PP 4_x_. For detailed explanations we refer to the caption of S1 Fig.
(TIF)

S5 Fig Evaluations plots for BR:FO. Upper left: HCA dendrogram suggests selection of four responder chronologies. Note, that cluster four only consists of five specimens. Upper right: *grbar*_*N*_ clearly indicates signal strength enhancement at the margins of the population. Mid left: PCGA extreme responder chronologies show strongly negative correlations with each other. Mid right: PCGA and HCA responder chronology signal correlations are comparably strong: see also Table 2. Lower left: Same as mid-left but here for the analyses where the minimum sample size was adjusted to match HCA minimum sample size. Lower right: Same as mid right but here for the analyses where the minimum sample size was adjusted to match HCA minimum sample size.
(TIF)

S6 Fig Evaluation plots for BR:TL. For detailed explanations we refer to S5 Fig.
(TIF)

S7 Fig Evaluation plots for DN:FO. For detailed explanations we refer to S5 Fig.
(TIF)

S8 Fig Evaluation plots for DN:TL. For detailed explanations we refer to S5 Fig.
(TIF)

S1 R Code (DOCX)

## Abbreviations

EPS: Expressed Population Signal
GR: Growth Ratio approach
HCA: Hierarchical Cluster Analysis
PCA: Principal Component Analysis
PCGA: Principal Component Gradient Analysis
PP: Pseudo-Populations
rbar: mean inter-series correlation
RRCSS: Relative Responder Chronology Signal Strength
RWS: Ring-Width Series
TR: TRend approach
