# Supplementary figures and images for: Tuning the Voices of a Choir: Detecting Ecological Gradients in Time-Series Populations
Source: PLoS One. 2016 Jul 28;11(7):e0158346. doi: 10.1371/journal.pone.0158346 (PMC4965043; doi:10.1371/journal.pone.0158346)

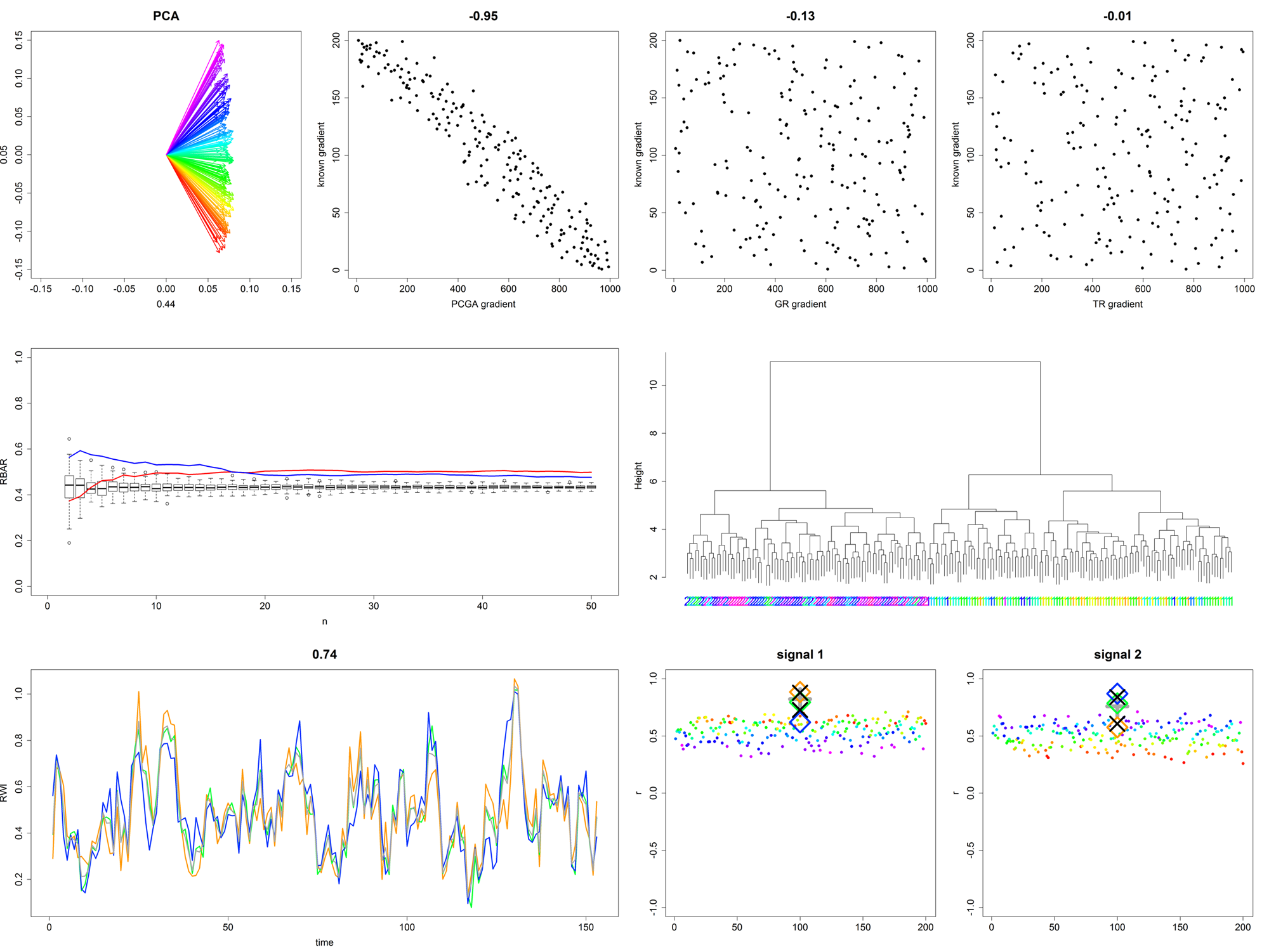

Supplement: S1 Fig — Upper left panel: Loadings of the PCA over RWS coloured according to the PCGA gradient. Axis labels refer to PC relative importance. Three upper right panels: Detected gradients plotted against known gradients for PCGA, GR, and TR, respectively. Headers of these panels reflect the correlation coefficient between detected and true gradient. Mid left: grbarN plotted against ExrbarN¯ along the population gradient for both population margins (red and blue lines). Both grbarN strongly suggest a population gradient as being well above ExrbarN¯. Mid right: HCA dendrogram suggested defining two responder chronologies which mostly correctly split RWS along the known gradient. Lower left: PCGA responder chronologies (colored curves) show a minimum correlation of 0.74 among each other. Two lower right panels: RWS to signal correlations (small dots coloured according to PCGA gradient) show a clear relationship with PCGA gradient. PCGA (coloured ‘◊’) and HCA (black ‘X’) responder chronologies show equal signal correlations of which the marginal responder chronologies express higher correlations than the population master (grey ‘+’). (TIF) [file pone.0158346.s001.tif]

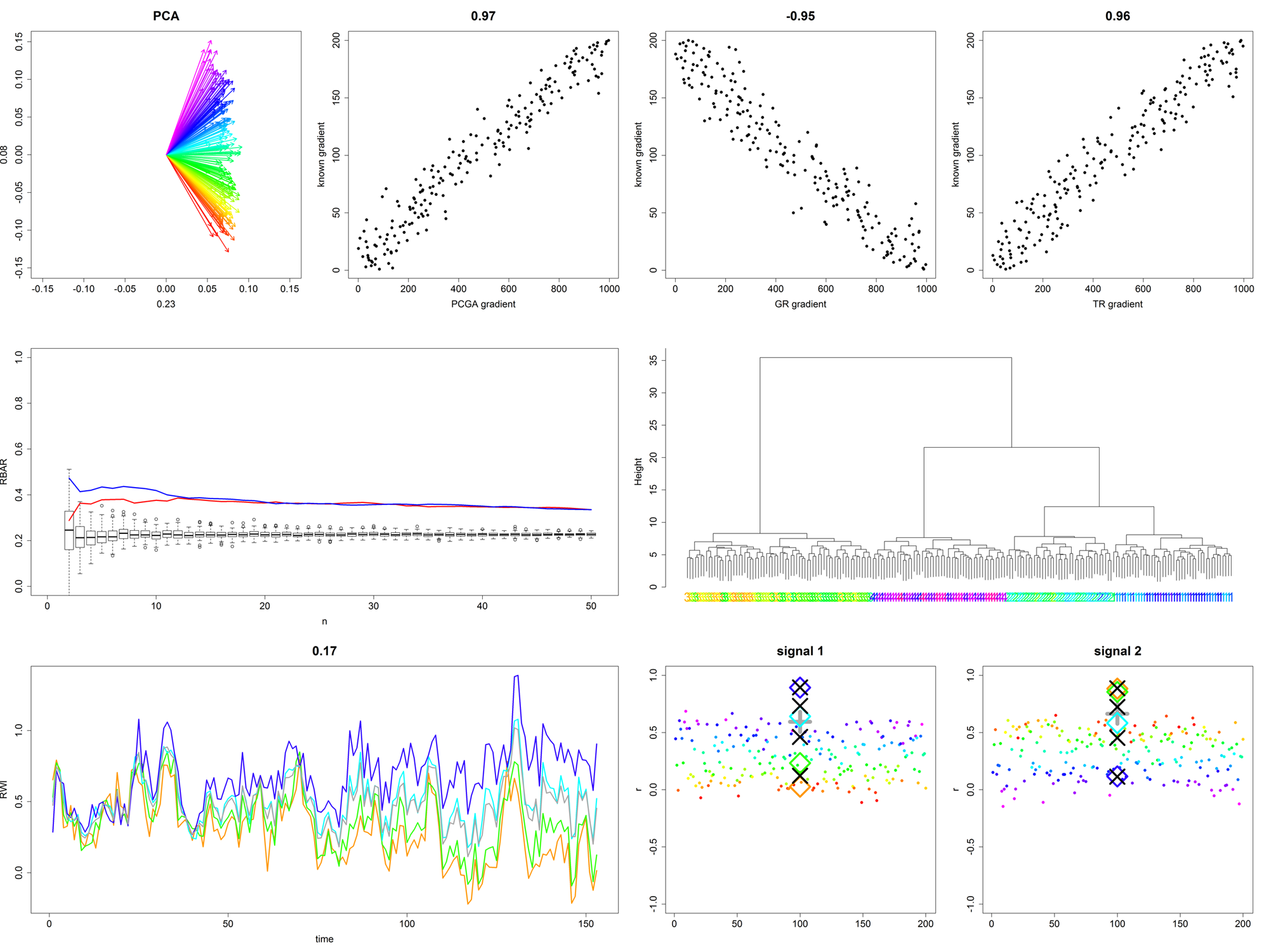

Supplement: S2 Fig — For detailed explanations we refer to the caption of S1 Fig. (TIF) [file pone.0158346.s002.tif]

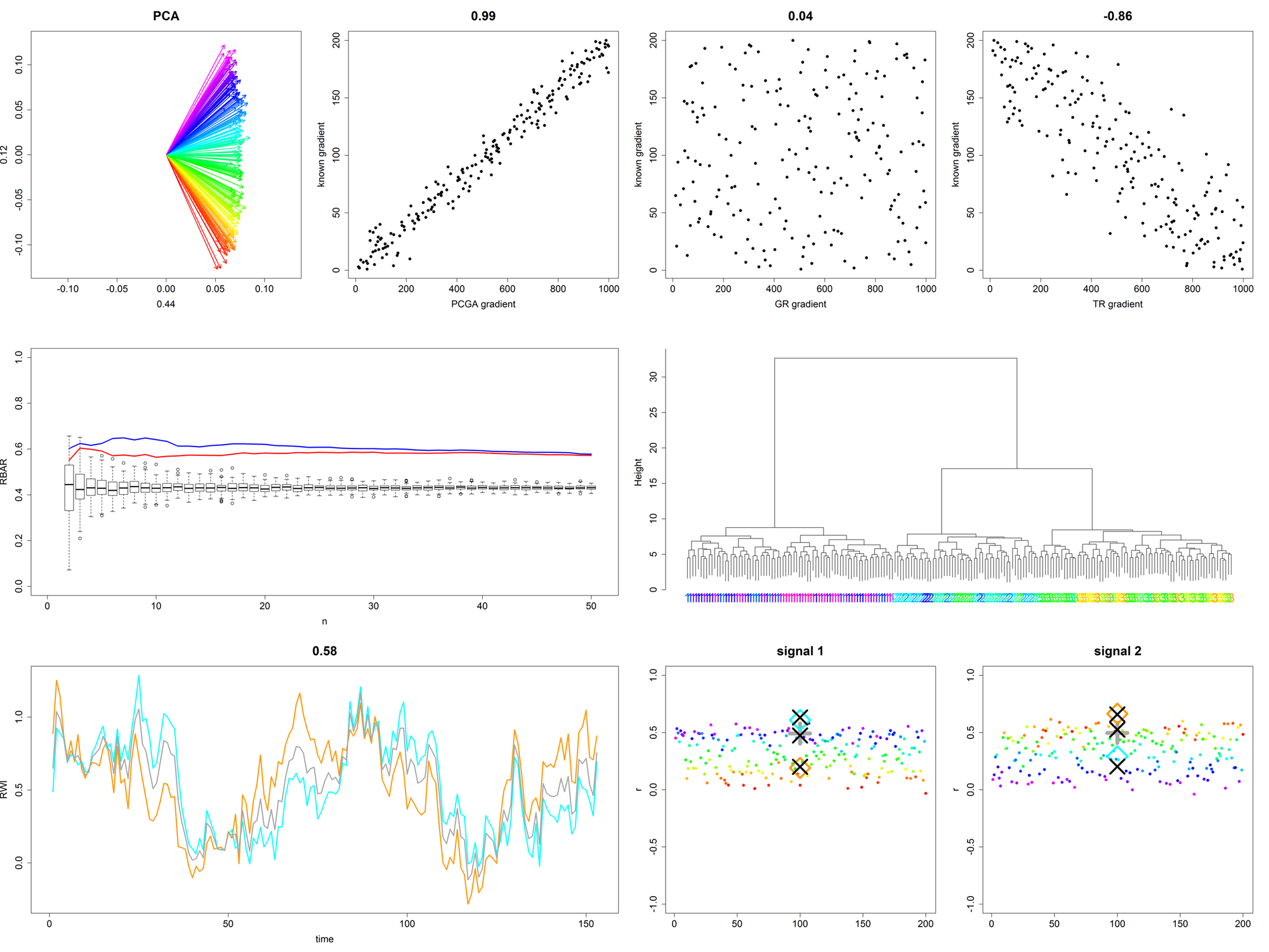

Supplement: S3 Fig — For detailed explanations we refer to the caption of S1 Fig. (TIF) [file pone.0158346.s003.tif]

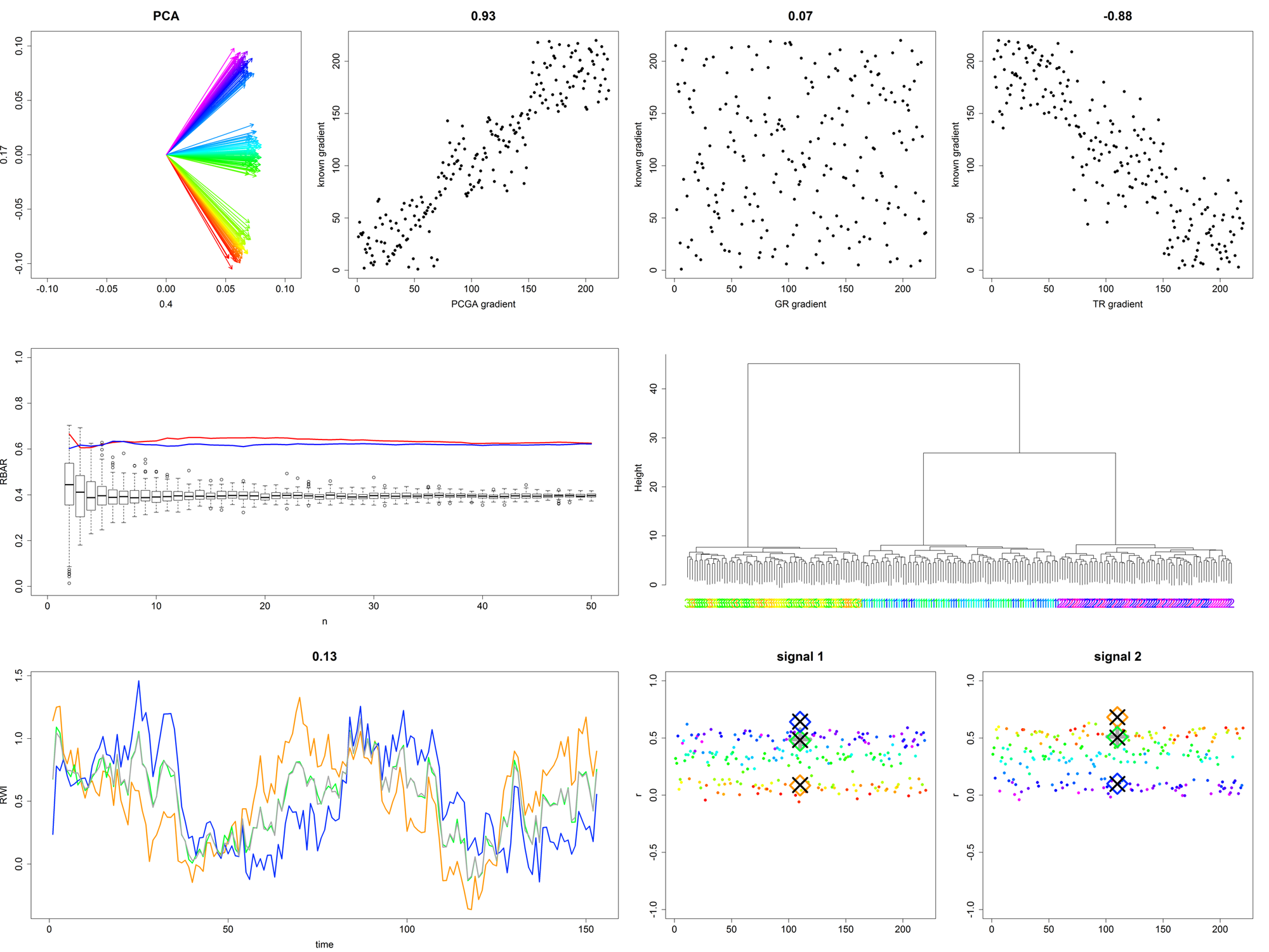

Supplement: S4 Fig — For detailed explanations we refer to the caption of S1 Fig. (TIF) [file pone.0158346.s004.tif]

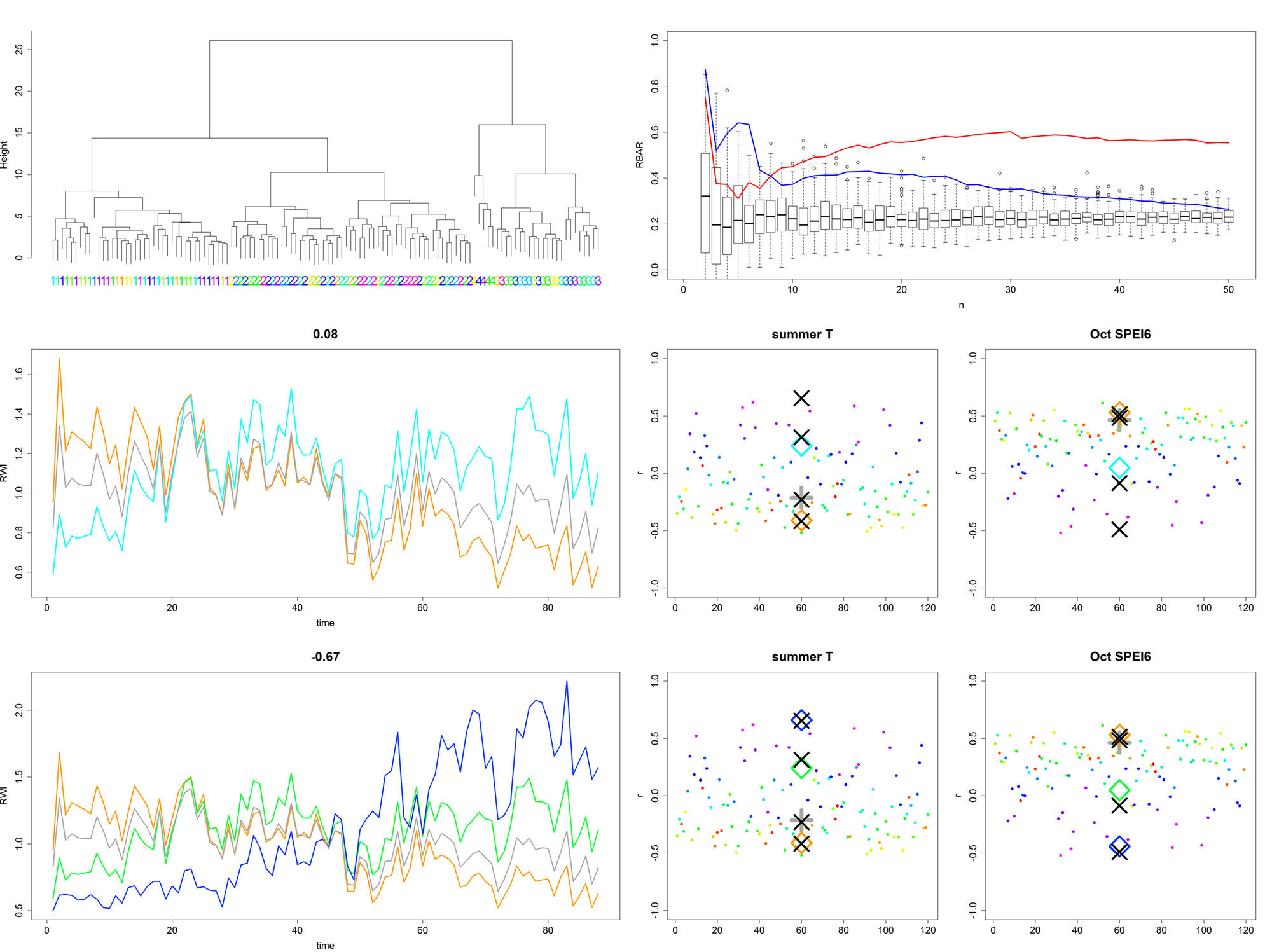

Supplement: S5 Fig — Upper left: HCA dendrogram suggests selection of four responder chronologies. Note, that cluster four only consists of five specimens. Upper right: grbarN clearly indicates signal strength enhancement at the margins of the population. Mid left: PCGA extreme responder chronologies show strongly negative correlations with each other. Mid right: PCGA and HCA responder chronology signal correlations are comparably strong: see also Table 2. Lower left: Same as mid-left but here for the analyses where the minimum sample size was adjusted to match HCA minimum sample size. Lower right: Same as mid right but here for the analyses where the minimum sample size was adjusted to match HCA minimum sample size. (TIF) [file pone.0158346.s005.tif]

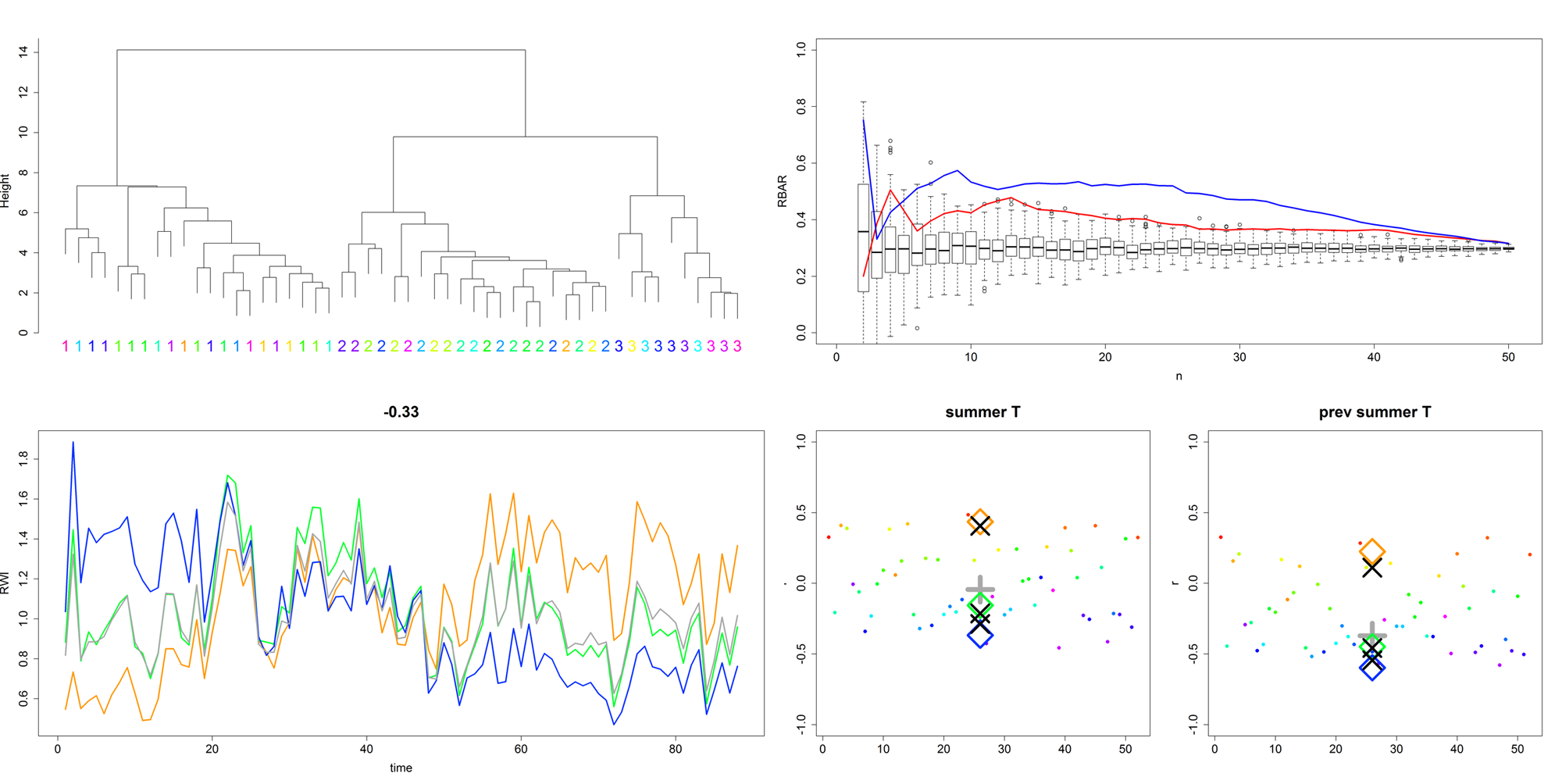

Supplement: S6 Fig — For detailed explanations we refer to S5 Fig. (TIF) [file pone.0158346.s006.tif]

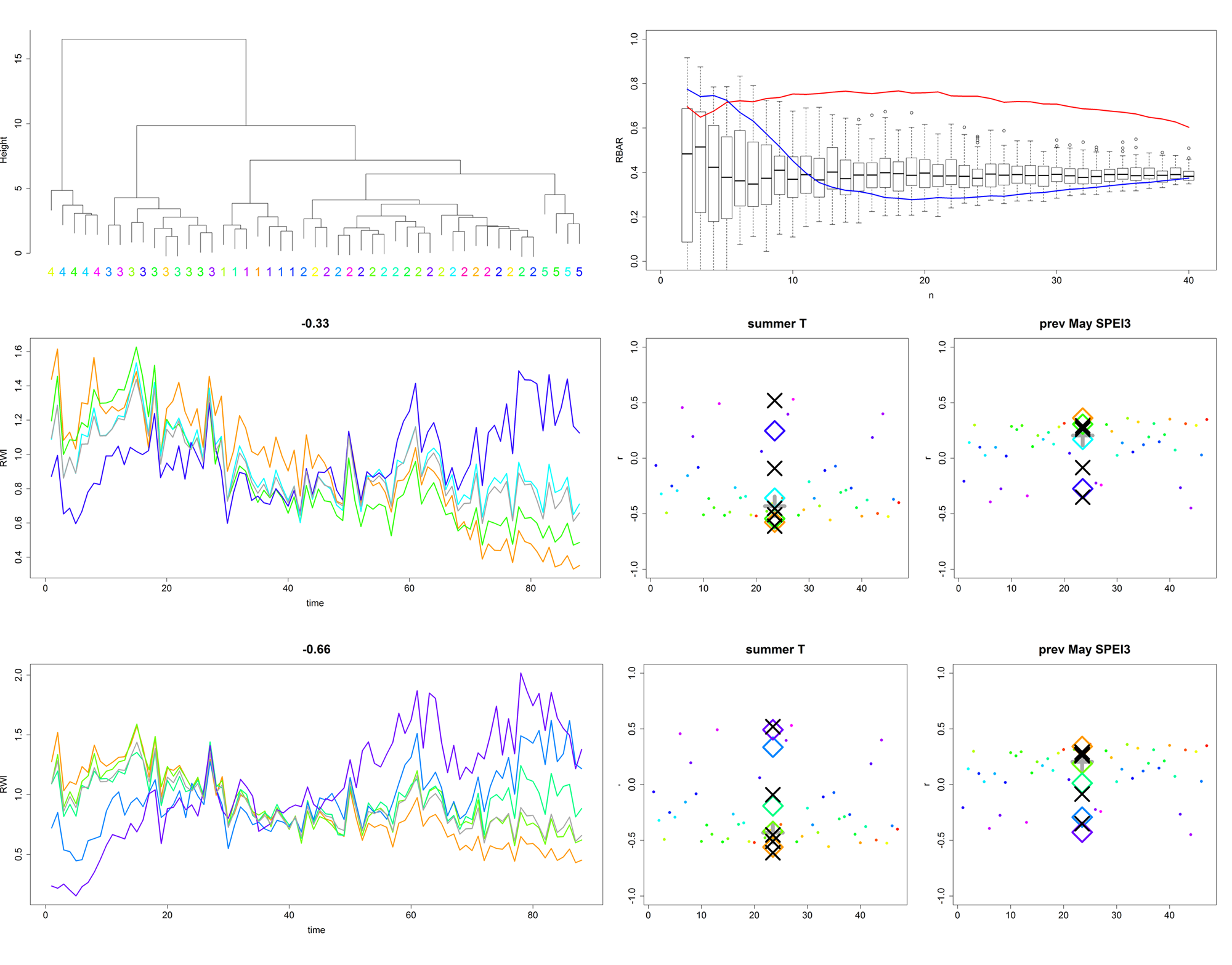

Supplement: S7 Fig — For detailed explanations we refer to S5 Fig. (TIF) [file pone.0158346.s007.tif]

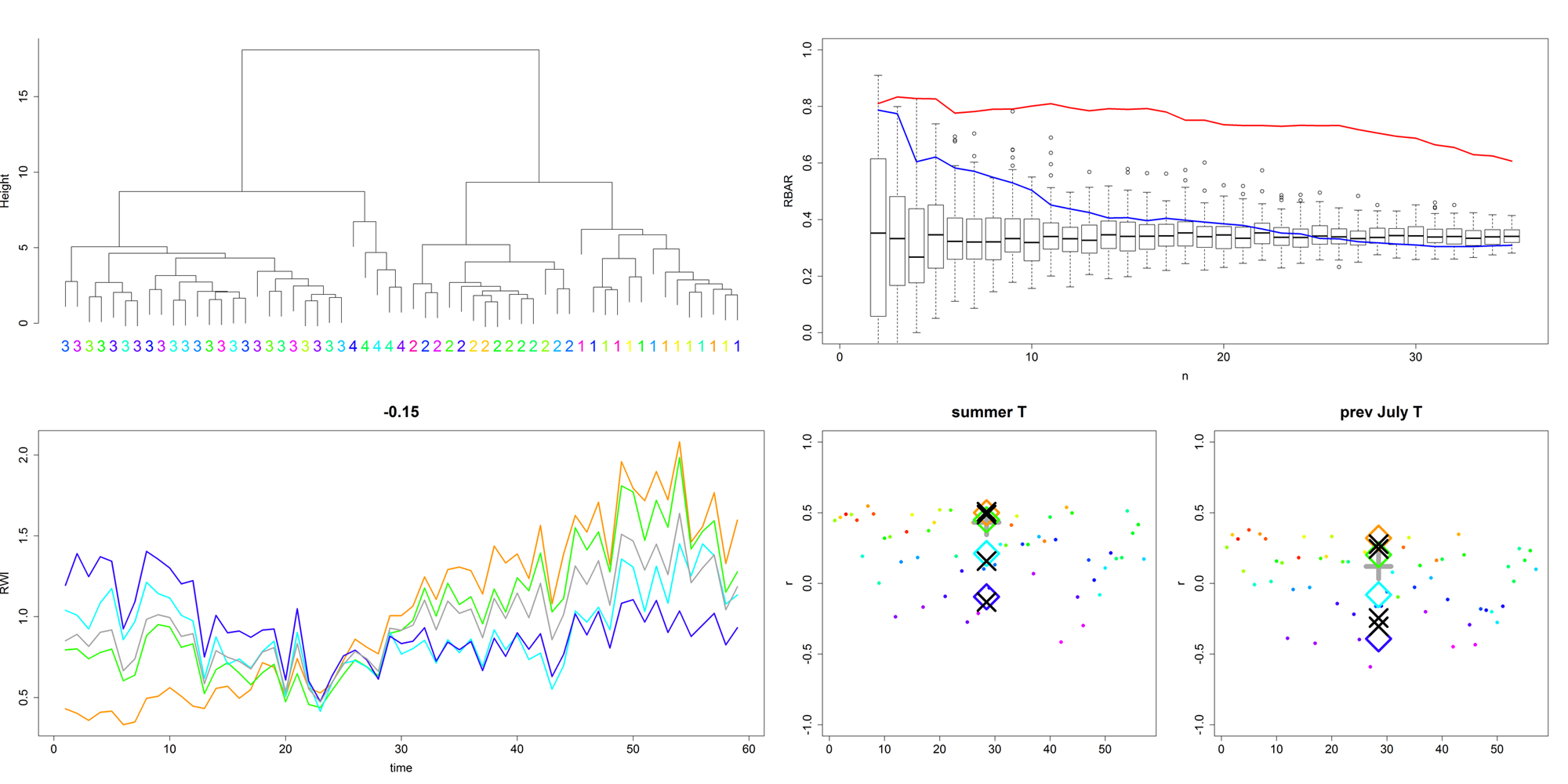

Supplement: S8 Fig — For detailed explanations we refer to S5 Fig. (TIF) [file pone.0158346.s008.tif]
