# Supplementary material for: Tuning the Voices of a Choir: Detecting Ecological Gradients in Time-Series Populations
Source: PLoS One. 2016 Jul 28;11(7):e0158346. doi: 10.1371/journal.pone.0158346 (PMC4965043; doi:10.1371/journal.pone.0158346)
Supplement: S1 R Code — (DOCX) [file pone.0158346.s009.docx]

**R code.**

###transforming axes loadings into polar coordinates of the rotation matrix

polar.trans<-function(matrix)

{

POL_MAT<-matrix(nrow=nrow(matrix),ncol=2)

for(i in 1:nrow(matrix))

{

POL_MAT[i,2]<-dist(rbind(c(0,0),matrix[i,1:2]),method="euclidean")

POL_MAT[i,1]<-atan2(matrix[i,2],matrix[i,1])

}

return(POL_MAT)

}

###PCGA gradient detection function

PCGA<-function(DATA)

{

PCA<-prcomp(DATA,scale=T)

IMP<-summary(PCA)$imp[,1:4]

POL_COORD<-polar.trans(PCA$rot[,1:2])

POL_COORD2<-polar.trans(-PCA$rot[,1:2])

if(max(dist(POL_COORD[,1]))>max(dist(POL_COORD2[,1])))

{

POL_COORD<-POL_COORD2

PCA$rot<--PCA$rot

}

SORT_POL_1<-vector(mode="numeric")

for(i in 1:nrow(POL_COORD))

{

SORT_POL_1[i]<-which(POL_COORD[,1]==sort(POL_COORD[,1])[i])

}

list(pca=PCA,imp=IMP,rank=SORT_POL_1,pol.coord=POL_COORD)

}
